# Abdominal Fat SIRT6 Expression and Its Relationship with Inflammatory and Metabolic Pathways in Pre-Diabetic Overweight Patients

**DOI:** 10.3390/ijms20051153

**Published:** 2019-03-06

**Authors:** Nunzia D’Onofrio, Gorizio Pieretti, Feliciano Ciccarelli, Antonio Gambardella, Nicola Passariello, Maria Rosaria Rizzo, Michelangela Barbieri, Raffaele Marfella, Gianfranco Nicoletti, Maria Luisa Balestrieri, Celestino Sardu

**Affiliations:** 1 Department of Precision Medicine, University of Campania “Luigi Vanvitelli”, via L. De Crecchio 7, 80138 Naples, Italy; nunzia.donofrio@unicampania.it; 2 Multidisciplinary Department of Medical, Surgical and Dental Specialties, University of Campania “Luigi Vanvitelli”, 80138 Naples, Italy; dott.goriziopieretti@gmail.com (G.P.); giovannifrancesco.nicoletti@unicampania.it (G.N.); 3 Breast Unit and Plastic Surgery, Villa dei Fiori Hospital, 80011 Naples, Italy; feliciano.ciccarelli@libero.it; 4 Department of Advanced Medical and Surgical Sciences, University of Campania “Luigi Vanvitelli”, 80138 Naples, Italy; antonio.gambardella@unicampania.it (A.G.); nicola.passariello@unicampania.it (N.P.); mariarosaria.rizzo@unicampania.it (M.R.R.); michelangela.barbieri@unicampania.it (M.B.); raffaele.marfella@unicampania.it (R.M.); celestino.sardu@unicampania.it (C.S.)

**Keywords:** sirtuin 6, obesity, pre-diabetes, NF-κB, PPAR-γ, SREBP-1

## Abstract

The role of sirtuin 6 (SIRT6) in adipose abdominal tissue of pre-diabetic (pre-DM) patients is poorly known. Here, we evaluated SIRT6 expression in visceral abdominal fat of obese pre-diabetic patients and the potential effects of metformin therapy. Results indicated that obese pre-DM subjects showed low SIRT6 protein expression and high expression of nuclear factor kappa-light-chain-enhancer of activated B cells (NF-κB), peroxisome proliferator-activated receptor gamma (PPAR-γ), and sterol regulatory element-binding transcription factor 1 (SREBP-1). Obese pre-DM patients showed high values of glucose, insulin resistance (HOMA-IR), C reactive protein (CRP), nitrotyrosine, tumor necrosis factor-α (TNF-α) and interleukin 6 (IL-6), and low values of insulin (*p* < 0.05). Of note, abdominal fat tissue of obese pre-DM patients treated with metformin therapy presented higher SIRT6 expression and lower NF-κB, PPAR-γ, and SREBP-1 expression levels compared to pre-DM control group. Collectively, results show that SIRT6 is involved in the inflammatory pathway of subcutaneous abdominal fat of obese pre-DM patients and its expression responds to metformin therapy.

## 1. Introduction

Visceral fat and superficial adipose tissue of obese patients express cytokines cross-talking with the cardiovascular system [1]. It has been recently reported that inflammatory/oxidative molecules and sirtuin 1 (SIRT1) protein expression in the abdominal fat tissue of pre-diabetic (pre-DM) obese patients negatively correlate with cardiac performance [2]. Moreover, reduced inflammation and oxidative burden have been observed in overweight pre-diabetic patients treated with metformin therapy in addition to the hypocaloric diet [2]. SIRT1, a member of the sirtuin family NAD+-dependent deacetylases, is a key regulator of adipose tissue function, mitochondrial biogenesis and adipocyte cell hypertrophy [1,2,3,4]. In addition to SIRT1, SIRT6 protects against inflammation, oxidative stress, and cardiovascular disease. SIRT6 regulates the activities of several non-histone proteins by lysine deacetylation, thus modulating the expression of target genes involved in the control of glucose homeostasis, energy metabolism, aging, oxidative stress, insulin resistance, lipids metabolism, and inflammation [5,6,7]. In particular, adipocyte-specific SIRT6 knockout mice showed an increased inflammation grade in the adipose tissue along with body weight increase and systemic insulin resistance [8]. In contrast, SIRT6 over expression resulted in the reduction of the fat mass, lowering LDL cholesterol and triglyceride levels, and improving glucose tolerance [8]. Moreover, SIRT6 knockout mice showed an increased expression of inflammatory genes, tumor necrosis factor-α (TNF-α), interleukin 6 (IL-6), and monocytes chemo attractive protein-1 (MCP-1) in both white and brown adipose tissues [9]. Actually, less is known about the specific molecular effectors regulated by SIRT6 in adipose tissue of obese pre-DM patients and the possible effect of metformin therapy. In this context, we aimed at investigating the possible role of SIRT6 in the abdominal fat of overweight pre-DM patients. To this end, protein expression levels of SIRT6 and its molecular targets were evaluated in adipose tissue samples from normoglycemic (NG), obese pre-DM, and obese pre-DM subjects under metformin therapy. Moreover, the possible correlation between plasma biomarkers of inflammation, glucose homeostasis, and lipid metabolism and the tissue expression of nuclear factor kappa-light-chain-enhancer of activated B cells (NF-κB) [10], PPAR-γ [11], and sterol regulatory element-binding transcription factor 1 (SREBP-1) [12] was investigated.

## 2. Results

### 2.1. Clinical Characteristics of the Study Population

At the enrollment, pre-DM patients presented higher values of intima-media thickness than obese NG patients (*p* < 0.05) (Table 1). Obese pre-DM patients (group 3) and obese pre-DM patients treated with metformin therapy (group 2) presented higher values of glucose (*p* < 0.05), lower values of insulin (*p* < 0.05), and higher values of HOMA-IR (*p* < 0.05) than NG obese patients (group 1) (Table 1). Moreover, group 2 and 3 patients showed higher values of creatinine (*p* < 0.05), inflammatory markers, such as CRP (*p* < 0.05), IL-6 (*p* < 0.05), TNF-α (*p* < 0.05), and nitrotyrosine (*p* < 0.05), than NG obese patients (group 1) (Table 1). The same trend was observed when pre-DM metformin users were compared to pre-DM non-metformin users (*p* < 0.05) (Table 1).

### 2.2. Abdominal Adipose Tissue Expression of SIRT6, NF-κB, PPAR-γ, and SREBP-1

**Figure 1:**
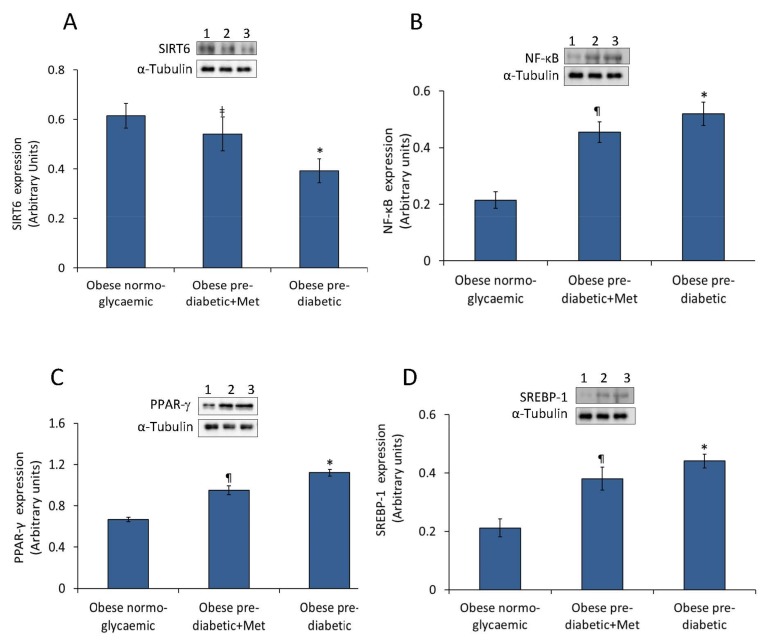
Protein expression levels of SIRT6, NF-κB, PPAR-γ and SREBP-1 in abdominal adipose tissue. (**A**) SIRT6, (**B**) NF-κB, (**C**) PPAR-γ, and (**D**) SREBP-1 protein levels determined by Western blot analysis of abdominal adipose tissue homogenates from obese normoglycaemic, obese pre-diabetic patients plus metformin (Met) and obese pre-diabetic patients treated with placebo. Inset, representative images of Western blot analysis. Lane 1, obese normoglycaemic patient; lane 2, obese pre-diabetic+ Met; lane 3, obese pre-diabetic + placebo. Data are mean ± SD. * *p* < 0.01 vs. obese normoglycemic patients; ^‡^ *p* < 0.01 vs. obese pre-diabetic + placebo; ^¶^ *p* < 0.05 vs. obese pre-diabetic + placebo.

Western blot analysis revealed that abdominal adipose tissue of obese pre-DM obese patients showed lower levels of SIRT6 and higher levels of NF-κB, PPAR-γ and SREBP-1 (Figure 1). Specifically, results indicated a significant down-regulation of SIRT6 protein expression in pre-DM subjects as compared to obese NG patients (*p* < 0.01). Obese pre-DM not-metformin users presented lower values of SIRT6 (*p* < 0.01 vs. NG) and higher values of NF-κB, PPAR-γ and SREBP-1 (*p* < 0.01 vs. NG) (Figure 1). Moreover, obese pre-DM under metformin therapy showed higher values of SIRT6 expression (*p* < 0.05) and lower values of NF-κB, PPAR-γ, and SREBP-1 protein levels (*p* < 0.05) compared to obese pre-DM not-metformin users (Figure 1). 

## 3. Discussion

Here, we provide a novel evidence on the involvement of SIRT6 in the inflammatory pathway occurring in the abdominal adipose tissue of obese pre-DM patients. Results indicated that obese pre-DM patients showed higher values of inflammatory and oxidative stress markers, and lower values of SIRT6 tissue protein expression than normoglycemic subjects, with a beneficial effect exerted by metformin therapy. Several studies have documented the anti-inflammatory properties of SIRT6 [6,13,14]. A decreased expression of SIRT6 has been reported in the atherosclerotic plaque of diabetic patients, with a concomitant expression of higher levels of inflammatory and oxidative stress markers [15]. SIRT6 activity is likely to be negatively regulated through reactive nitrogen species-mediated tyrosine nitration during oxidative stress [16]. Here, the expression of SIRT6 in adipose tissue is related to high serum values of nitrotyrosine in obese pre-DM compared to obese NG, and in obese pre-DM non-metformin users as compared to obese pre-DM metformin users (*p* < 0.05). However, a regulatory effect on SIRT6 expression might be the main determinant of oxidative stress and nitrotyrosine reduction in pre-DM receiving an anti-oxidative drug therapy with metformin [17]. The anti-inflammatory role of SIRT6 is supported by the observation that SIRT6 promotes the silencing of NF-κB target genes through deacetylation of H3K9 at target gene promoters, decreasing NF-κB-dependent apoptosis and senescence [18]. It has been reported that SIRT6 deficiency in SIRT6 null mice (SIRT6^−/−^) determined the activation of pro-inflammatory genes, such as MCP-1, IL-6, and TNF-α [14]. Here, we observed an increased expression of adipose tissue NF-κB protein levels in obese pre-DM patients compared to obese NG patients (*p* < 0.01), along with increased circulating levels of IL-6, TNF-α and CRP (*p* < 0.05). This effect might be linked to a lower tissue expression of SIRT6. Intriguingly, metformin therapy decreased NF-κB (*p* < 0.05), IL-6, TNF-α and CRP proteins levels (*p* < 0.05) in adipose tissue of obese pre-DM patients. Moreover, obese pre-DM metformin patients showed a trend of increase in triglyceride values compared to pre-DM placebo patients (1.89 ± 0.44 vs. 1.83 ± 0.54 mmol/L), without statistical significance. Despite the evidence of well-matched baseline clinical variables of the study population, 8 patients in the pre-DM metformin group were under oral hypolipidemic drugs compared to 9 patients in the pre-DM treated placebo group and, although this was not statistically significant, it might influence the triglyceride values.

Obese pre-DM patients showed glucose intolerance, more evident in obese pre-DM patients without metformin therapy, higher glucose blood levels, and insulin resistance (*p* < 0.05), whereas obese pre-DM patients receiving metformin therapy showed an improved glucose metabolism (*p* < 0.05). Of interest is the observation that abdominal adipose tissue from obese pre-DM patients, expressing lower values of SIRT6, showed higher values of PPAR-γ and SREBP-1 when compared to NG obese patients (*p* < 0.01). Intriguingly, decreased of PPAR-γ expression levels were observed when obese pre-DM metformin users were compared to obese pre-DM non-metformin users (*p* < 0.05). These results support the well-known role of SIRT6 in glucose metabolism and the link between this sirtuin and PPAR-γ pathways [19,20,21,22,23,24]. In this context, it is important to highlight the association between SIRT6 and lipids metabolism [25,26,27,28,29,30,31], as this sirtuin is implied in the control of lipid mobilization, fatty acid uptake and thermogenesis in the adipose tissue [25,26,27,28,29,30,31]. Moreover, mice over-expressing SIRT6 showed lower low-density lipoprotein cholesterol levels via the lipogenic transcription factors SREBP-1 and SREBP-2 [28].

Overall, results of this study provide a clinical evidence of the role of SIRT6 role in pre-DM insulin resistance and inflammation in the adipose tissue of obese patients. Although metformin therapy seems to regulate SIRT6 metabolic pathways, the molecular mechanisms through which it acts remain to be fully elucidated before considering SIRT6 as a novel molecular target in preventing adipose tissue inflammation. Limitations of this study are: (i) the small sample size of pre-diabetics obese which affects the study results; (ii) the lack of animal or cellular models to test the human study results obtained by peripheral blood analysis and by direct analysis of samples by abdominal fat tissue biopsy; (iii) no baseline data before metformin or placebo treatment are reported, so the evidence cannot rule out the difference of individuals, especially insulin, glucose, lipids, and inflammatory markers. Thus, further long-term studies in a larger population of pre-diabetics obese will be necessary to confirm these findings and to determine whether the metformin-induced regulation of SIRT6 added to abdominoplastic surgery and hypocaloric diet translates into a reduced incidence of cardiovascular disease in obese pre-diabetic patients.

## 4. Materials and Methods

### 4.1. Research Design

In this prospective study, conducted from January 2015 to January 2018 at the University of Campania “Luigi Vanvitelli”, 50 obese patients with standard indications to receive an abdominoplastic surgery were enrolled [32]. Obesity was diagnosed as body mass index (BMI) > 30 [32]. All 50 patients underwent abdominoplastic surgery and received a hypocaloric diet. The mean recommended daily caloric intake was 1300 kcal, ranging from 1250 to 1350 kcal. The recommended composition of the dietary regimen was 55% carbohydrates, 30% lipid, and 15% protein. Forty obese patients had pre-diabetes according to international guidelines diagnostic criteria [33,34]. Pre-DM was diagnosed by evidence of fasting plasma glucose of ≥5.6 mmol/L but <7.0 mmol/L (100–125 mg/dL, impaired fasting glucose [IFG]), a 2-h glucose of ≥7.8 mmol/L but <11.1 mmol/L during a 75 g oral glucose tolerance test (GTT) (140–199 mg/dL; impaired glucose tolerance [IGT]), or a plasma hemoglobin (Hb) A1c of ≥5.7% but <6.5% (14). Pre-DM patients were divided in two groups: group 2 (*n* = 16) under metformin therapy (metformin users) and group 3 (*n* = 16) non-metformin users. The pre-DM patients in group 2 were in chronic treatment with metformin 850 mg twice a day. Eighteen patients were obese NG. All study groups volunteered for repeated clinical evaluations and laboratory analyses as well as echocardiography. Exclusion study criteria were diagnosis of type 2 diabetes, cardiovascular disease, psychiatric problems, a history of alcohol abuse, smoking, or insulin therapy assumption. All patients had normal results in terms of laboratory data (urea nitrogen, creatinine, electrolytes, liver function tests, uric acid, thyroxin, and complete blood count), chest x-rays, and electrocardiograms. Each patient provided informed written consent to participate in this study, which was approved by the institutional committee of ethical practice of our institution. The patients subscribed a separate informed consent to undergo abdominoplasty. Clinical research trial number: NCT03491241.

### 4.2. Anthropometrics Parameters

In all patients, height, weight, and BMI, calculated as weight in kilograms divided by the square of height in meters [1], were measured. Waist hip ratio (WHR) was calculated as waist circumference in centimeters divided by hip circumference in centimeters, and as index of central obesity [1]. As previously reported [1], insulin blood levels and the homeostasis model for the assessment of insulin resistance (HOMA-IR) were evaluated.

### 4.3. Blood Sample Analyses

Serum samples for cytokine levels were stored at temperature under 80 °C until assayed, as previously reported [2]. Serum concentrations of TNF-α, IL-6, and nitrotyrosine were determined in duplicate using a highly sensitive quantitative sandwich enzyme assay (ELISA, Quantikine HS; R and D Systems, Minneapolis, MN, USA). Venous blood samples were drawn for nitrotyrosine evaluation. Nitrotyrosine plasma concentration was determined, after an overnight fast, at breakfast time, and before the sensor insertion. Assays of serum total cholesterol and high-density lipoprotein cholesterol, triglyceride, and glucose levels were performed in the hospital’s chemistry laboratory. Plasma insulin levels were assayed by radioimmunoassay (Ares, Serono, Italy). Insulin resistance in the fasting state was assessed with homeostasis model assessment (HOMA) and calculated with the following formula: fasting plasma glucose (millimoles per liter) times fasting serum insulin (microunits per milliliter) divided by 25, as described previously [1,2].

### 4.4. Abdominal Dermolipectomy

Patients underwent conventional abdominoplasty surgical procedure, with umbiliculum transposition and cutaneous adipose mass tissue excision ranging from 200 g, as previously reported [1]. Patients were mobilized for 24 h after surgery. Anti-inflammatory therapy (non-steroidal anti-inflammatory drugs) was suspended after 48 h and were discharged 72 h following with antibiotic therapy.

### 4.5. Analysis of Adipose Tissue

After surgery, the specimens were cut parallel to the long axis into four different parts for the different works-ups. An aliquot was frozen in liquid nitrogen for the following enzyme-linked immunosorbent assay analysis, according to manufacturer’s instructions. 

### 4.6. Western Blot Analysis

The detection of SIRT6, NF-κB, PPAR-γ, and SREBP-1 expression levels was carried out in adipose abdominal tissue. Cutaneous adipose mass samples (100 mg) from obese NG and obese pre-DM patients were cut into small pieces before starting homogenization process performed by adding to tissues 500 µL of 2D lysis buffer (7 mM urea, 2 mM thiourea, 4% CHAPS [3-([3-cholamidopropyl] dimethylammonio)-1-propane sulfonate] buffer, 30 mM Tris-HCl, pH 8.8. Tissues were homogenized with a Precellys-24 system (Bertin Technologies, Montignyle-Bretonneux, France) and centrifuged at 800× *g* for 10 min at 4 °C to collect the supernatant. Protein extracts (50 µg) were separated by SDS-polyacrylamide gel electrophoresis (10%) and then transferred to nitrocellulose membranes by using the Trans-Blot Turbo Transfer System ((Bio-Rad Laboratories, Milan, Italy). After incubation with 5% *w*/*v* skim milk for 2 h, blots were incubated with primary antibodies against SIRT6 (1:1000) (rabbit polyclonal, ab62739, Abcam, Cambridge, UK), NF-κB (1:1000) (rabbit monoclonal, C22B4, Cell Signaling Technology, Danvers, MA), PPAR-γ (1:1000) (rabbit polyclonal, ab59256, Abcam, Cambridge, UK) and SREBP-1 (1:1000) (rabbit polyclonal, bs-1402R, Bioss, MA, USA). After incubation with secondary antibody (1:10,000), membranes were washed three times and immunoreactive bands were detected by the enhanced chemiluminescence kit (Immobilon Western, Chemiluminescent HRP Substrate, Millipore, Billerica, MA, USA) and analyzed by using Image Lab 5.2.1, Molecular Imager ChemiDoc XRS Imaging system (Bio-Rad Laboratories, Milan, Italy). Antibody against α-tubulin (1:1000) (mouse monoclonal, 3873, Cell Signaling Technology, Danvers, MA, USA) was used for protein expression normalization.

### 4.7. Statistical Analysis

Data were presented as group mean ± SD. One-way analysis of variance (ANOVA) was used to compare baseline data, followed by Scheffe’s test for pairwise comparisons. Simple and partial correlation were used to evaluate relationships between variables. *p* < 0.05 was considered significant. Statistical analysis was performed using the SPSS software package for Windows 17.0 (SPSS Inc., Chicago, IL, USA).

## Figures and Tables

**Table 1:** Clinical characteristics of study population. Study variables (biohumoral markers, echocardiographic parameters, fasting glucose, insulin, insulin resistance, lipid values and drug therapy) of study population of 50 patients divided in three groups at baseline: group 1, obese normoglycemic patients (NG) treated with hypocaloric diet (*n* = 18); group 2, pre-diabetic (pre-DM) obese patients treated with hypocaloric diet added to metformin (*n* = 16); group 3, pre-DM obese patients treated with hypocaloric diet added to placebo (*n* = 16). * *p* < 0.05 for the comparison of group 1 vs. group 2; ** *p* < 0.05 for the comparison of group 1 vs. group 3; *** *p* < 0.05 for the comparison of group 2 vs. group 3. The symbol/indicates a value not statistically significant.

Study Variables	Obese NG (*n* = 18)	Obese Pre-DM + Metformin (*n* = 16)	Obese Pre-DM + Placebo (*n* = 16)	*p* Value
**Baseline**				
***Clinical variables***				
Age	39.0 ± 8	40.5 ± 7	40.5 ± 6	/
Male (%)	5 (27.8)	5 (25)	6 (30)	/
BMI (kg/m^2^)	33.7 ± 2.4	33.1 ± 2.7	33.5 ± 2.6	/
Systolic arterial pressure (mmHg)	126 ± 10.3	133 ± 11	129 ± 12	/
Diastolic arterial pressure (mmHg)	85 ± 2.1	82 ± 2.3	84 ± 2.1	/
Heart rate (beats for minute)	69 ± 8	72 ± 9	72 ± 10	/
WHR	0.91 ± 0.001	0.91 ± 0.006	0.91 ± 0.005	/
HOMA-IR	4.1 ± 0.28	4.7 ± 0.72	4.9 ± 0.68	<0.05 *, <0.05 **
Insulin (µU/mL)	22.6 ± 1.9	20.9 ± 1.6	20.1 ± 1.8	<0.05 *, <0.05 **
Glucose (mmol/L)	5.34 ± 0.57	5.81 ± 0.18	6.73 ± 0.24	<0.05 *, <0.05 **, <0.05 ***
Cholesterol (mmol/L)	4.66 ± 1.02	4.33 ± 0.86	4.51 ± 0.88	/
HDL (mmol/L)	1.78 ± 0.41	1.73 ± 0.44	1.83 ± 0.39	/
LDL (mmol/L)	3.17 ± 0.59	3.31 ± 0.61	3.33 ± 0.57	/
Triglycerides (mmol/L)	1.61 ± 0.31	1.89 ± 0.44	1.83 ± 0.54	/
Creatinine (mmol/L)	78.3 ± 2.6	98.6 ± 4.4	101.2 ± 3.5	<0.05 *, <0.05 **
***Biohumoral inflammatory markers***				
CRP (mmol/L)	0.85 ± 0.38	0.97 ± 0.48	1.03 ± 0.43	<0.05 *, <0.05 **
IL-6 (pg/mL)	3.53 ± 0.43	3.81 ± 0.45	4.10 ± 0.39	<0.05 *, <0.05 **
TNF-α (pg/mL)	5.51 ± 1.09	6.19 ± 0.59	6.75 ± 0.53	<0.05 *, <0.05 **
Nitrotyrosine (nmol/L)	1.211 ± 0.205	3.283 ± 0.712	5.309 ± 0.651	<0.05 *, <0.05 **, <0.05 ***
***Adipose tissue markers***				
SIRT6 (arbitrary units)	1.14 ± 0.18	1.05 ± 0.14	0.94 ± 0.12	<0.05 *, <0.05 **, <0.05 ***
NF-κB (arbitrary units)	0.91 ± 0.06	1.02 ± 0.12	1.10 ± 0.09	<0.05 *, <0.05 **, <0.05 ***
PPAR-γ (arbitrary units)	0.67 ± 0.03	0.99 ± 0.04	1.07 ± 0.03	<0.05 *, <0.05 **, <0.05 ***
SREBP-1 (arbitrary units)	0.21 ± 0.03	0.39 ± 0.04	0.44 ± 0.02	<0.05 *, <0.05 **, <0.05 ***
***Echocardiographic parameters***				
Intima-media thickness	0.85 ± 0.14	1.01 ± 0.15	1.03 ± 0.18	<0.05 *, <0.05 **
LVTDd (mm)	54 ± 4.6	56 ± 3.8	55 ± 4.1	/
LVTSd (mm)	31 ± 6.7	34 ± 4.4	32 ± 4.8	/
LVEF (%)	53 ± 6	54 ± 6	54 ± 7	/
LAD (mm)	42 ± 2	45 ± 6	43 ± 6	/
Septum (mm)	13.5 ± 2.6	14 ± 2.5	14 ± 2.2	/
Posterior wall (mm)	11 ± 1	11 ± 1.5	11 ± 1	/
MPI	0.57 ± 0.03	0.58 ± 0.03	0.57 ± 0.03	/
LV mass (g)	203.7 ± 48.4	192.5 ± 49.5	191.7 ± 49.7	/
LV mass/BSA (g/m^2^)	90.13 ± 21.42	84.06 ± 21.62	82.62 ± 21.42	/
LV mass/h (m^2^)	72.23 ± 17.16	69 ± 17.75	67.03 ± 17.38	/
***Drug therapy***				
ACE inhibitors (%)	9 (50)	8 (50)	9 (56.2)	/
ARS blockers (%)	5 (28)	5 (31.2)	5 (31.2)	/
Calcium channels blockers (%)	2 (11.1)	2 (12.5)	2 (12.5)	/
Loop diuretics (%)	2 (11.1)	2 (12.5)	2 (12.5)	/
Metformin (%)	0	16 (100%)	0	
Statin (%)	7 (39)	8 (50)	9 (56.2)	/
Thiazides (%)	5 (27.8)	5 (31.2)	6 (37.5)	/

Abbreviations: ACE, angiotensin converting enzyme; ARS, angiotensin-renin system; CRP, C reactive protein; h, height; HOMA-IR, homeostasis model for the assessment of insulin resistance; IL6, interleukin 6; LAD, left atrium diameter; LV, left ventricle; LVEF, left ventricle ejection fraction; LVTDd, left ventricle telediastolic diameter; LVTDs, left ventricle telesystolic diameter; MPI, myocardium performance index; NG: normoglycemic; pre-DM: prediabetics; SIRT6, sirtuin 6; TNF-α tumor necrosis factor-α; WHR, waist hip ratio.

## Abbreviations

CRP	C reactive protein
IL-6	Interleukin 6
LVEF	Left ventricle ejection fraction
MPI	Myocardial performance index
NF-κB	Nuclear factor kappa-light-chain-enhancer of activated B cells
NG	Normoglycemic
PPAR-γ	Peroxisome proliferator-activated receptor gamma
pre-DM	Pre-diabetic
SIRT1	Sirtuin 1
SIRT6	Sirtuin 6
SREBP-1	Sterol regulatory element-binding transcription factor-1
TNF-α	Tumor necrosis factor-α
